# Microbial etiology of pneumonia in patients with decreased renal function

**DOI:** 10.1371/journal.pone.0216367

**Published:** 2019-05-09

**Authors:** Chun-you Chen, Shih-chang Hsu, Hui-ling Hsieh, Chi-won Suk, Yuan-pin Hsu, Yuh-mou Sue, Tso-Hsiao Chen, Feng-yen Lin, Chun-ming Shih, Jaw-wen Chen, Shing-jong Lin, Po-hsun Huang, Chung-te Liu

**Affiliations:** 1 Department of Radiation Oncology, Wan Fang Hospital, Taipei Medical University, Taipei, Taiwan; 2 Emergency Department, Department of Emergency and Critical Medicine, Wan Fang Hospital, Taipei Medical University, Taipei, Taiwan; 3 Department of Emergency Medicine, School of Medicine, College of Medicine, Taipei Medical University, Taipei, Taiwan; 4 Division of Nephrology, Department of Internal Medicine, Wan Fang Hospital, Taipei Medical University, Taipei, Taiwan; 5 Graduate Institute of Medical Science, National Defense Medical Center, Taipei, Taiwan; 6 Division of Pulmonary Medicine, Department of Internal Medicine, Wan Fang Hospital, Taipei Medical University, Taipei, Taiwan; 7 Graduate Institute of Clinical Medicine, College of Medicine, Taipei Medical University, Taipei, Taiwan; 8 Department of Internal Medicine, School of Medicine, College of Medicine, Taipei Medical University, Taipei, Taiwan; 9 Division of Cardiology and Cardiovascular Research Center, Department of Internal Medicine, Taipei Medical University Hospital, Taipei, Taiwan; 10 Division of Cardiology, Department of Medicine, Taipei Veterans General Hospital, Taipei, Taiwan; 11 Cardiovascular Research Center, National Yang-Ming University, Taipei, Taiwan; 12 Department of Medical Research, Taipei Veterans General Hospital, Taipei, Taiwan; 13 Institute of Pharmacology, National Yang-Ming University, Taipei, Taiwan; 14 Institute of Clinical Medicine, National Yang-Ming University, Taipei, Taiwan; 15 Board of Directors, Taipei Medical University, Taipei, Taiwan; University of Mississippi Medical Center, UNITED STATES

## Abstract

**Background:**

Patients with renal impairment have altered immunity, which might cause vulnerability to specific pathogens and worsen pneumonia-related outcomes. Nonetheless, the microbiological features of pneumonia in patients with decreased renal function remain unknown.

**Methods:**

Therefore, we conducted a retrospective cohort study enrolling adult patients hospitalized with pneumonia to assess this knowledge gap. The baseline estimated glomerular filtration rate (eGFR) and first sputum microbiology during hospitalization were used for statistical analyses.

**Results:**

Overall, 1554 patients hospitalized with pneumonia (mean age, 76.1 ± 16.7) were included, and 162 patients had died at the end of hospitalization. The cutoff eGFR value predicting mortality was <55 mL/min/1.73 m^2^, which defined decreased renal function in this study. Patients with decreased renal function demonstrated a significantly higher risk of fungi and *Staphylococcus aureus* (*S*. *aureus*) infection. On the other hand, this group of patients showed significantly higher neutrophil-to-lymphocyte ratio (NLR), which associated with higher mortality. Additionally, patients with *S*. *aureus* had a significantly lower eGFR, lymphocyte count and a higher NLR.

**Conclusions:**

These findings suggested the altered immunity and vulnerability to *S*. *aureus* infection in patients with decreased renal function, which may be the underlying cause of worse outcomes of pneumonia in this group of patients.

## Introduction

Pneumonia is a major cause of death worldwide [1–2]. Based on the report of the Global Burden of Disease Study, lower respiratory tract infection was the second leading cause of death in 2013, accounting for 3.85% of total deaths [3]. With a global incidence of 1.5–14 per 1000 person-years [4–6], pneumonia-related costs are also a serious burden to healthcare [7–9]. As such, the treatment of pneumonia remains a major issue. In patients with chronic kidney disease (CKD), pneumonia is a significant cause of infection-related hospitalization [10–12]. Several studies have demonstrated an increased risk of pneumonia-related hospitalization and mortality in patients with CKD [13–15]. Moreover, in patients hospitalized with pneumonia, acute kidney injury (AKI) is associated with adverse outcomes [16–17]. These findings indicate the association between decreased renal function and adverse outcomes for pneumonia.

The microbiological aspects of disease are an essential part of the treatment of pneumonia [18–19]. Generally, *Streptococcus pneumoniae* is the most common pathogen that causes this disease, contributing to 12–68% and 10–15% of cases in Europe [20] and the United States [21–23], respectively. Other common pathogens include *Haemophilus influenzae*, *Staphylococcus aureus*, *Moraxella catarrhalis*, and *Pseudomonas aeruginosa* [24–26]. However, for patients with decreased renal function, the microbiologic characteristics of pneumonia remain unknown. Disclosing such microbiologic characteristics might yield improved outcomes for pneumonia patients with renal impairment.

Previous studies indicated worse pneumonia-related outcomes in patients with decreased renal function, which might be explained by suboptimal immunity in this population. Indeed, altered immunity was demonstrated in patients with end-stage renal disease (ESRD) [27–28]. However, the association between altered immunity and outcomes of infection in patients with decreased renal function had not been defined. Hence, the investigation of immune cell profiles might represent a strategy to reveal the association between suboptimal immunity and outcome for patients with pneumonia and decreased renal function.

As such, disclosing the microbiological features and immune cell profiles might be helpful to improve our understanding and the outcome of pneumonia patients with decreased renal function. To that end, we conducted a retrospective study of patients who were hospitalized for pneumonia.

## Materials and methods

### Study design and subjects

In the present study, adult patients hospitalized with pneumonia at Wan Fang Hospital, Taipei Medical University between January 2013 and December 2015 were included. All patients were admitted to the emergency department with pneumonia and were hospitalized. The primary discharge diagnosis was community-acquired or healthcare-associated pneumonia. Patients < 20 years of age were excluded. This study was approved by the ethics committee and Institutional Review Board of Taipei Medical University (N201805061) and the informed consent was waived. The present study was also conducted in accordance with the tenets of the 1975 Declaration of Helsinki, as revised in 2000.

### Definition of covariates and outcomes

The presence of CKD was defined as an estimated glomerular filtration rate (eGFR) less than 60 mL/min/1.73m^2^ for more than 3 months before the indexed hospitalization. AKI was defined by increase in serum creatinine by ≥ 0.3 mg/dL within 48 hours or increase in serum creatinine to ≥ 1.5 times baseline within the prior 7 days, based on KDIGO Clinical Practice Guideline for Acute Kidney Injury suggested in 2012 [29]. For a patient lacking past serum creatinine values, CKD and AKI were defined according to the discharge diagnosis. Patients with ESRD were included in the present study and were defined as initiation of maintenance dialysis before the indexed hospitalization by reviewing medical records. Diabetes mellitus (DM), congestive heart failure (CHF), and chronic obstructive pulmonary disease (COPD) were defined according to the discharge diagnoses. The baseline laboratory data obtained at the emergency department were used for statistical analyses. In the present study, eGFR was calculated by the equation suggested by the Chronic Kidney Disease Epidemiology Collaboration in 2009 [30]. Of note, for patients with ESRD, the eGFR was uniformly regarded as 5 mL/min/m^2^ for statistical analyses for two reasons. First, eGFR calculated from serum creatinine levels might be disturbed by hemodialysis sessions before the visit to the emergency department, confounding the statistical analyses. Second, eGFR < 5 mL/min/m^2^ is the criteria for the initiation of maintenance dialysis, as defined by the National Institute Insurance of Taiwan.

For analysis of immune cell profiles, absolute cell counts expressed in cell/mm^3^ [31], along with the neutrophil-to-lymphocyte ratio (NLR) and the monocyte-to-lymphocyte ratio (MLR) were used. For microbiological analyses, the pathogen was defined as the bacteria cultured from the first sputum specimen upon the indexed hospitalization. Patients without available microbiology reports were excluded from these analyses. The outcome of the present study was in-hospital mortality defined as death at the end of the indexed hospitalization.

### Statistical analysis

Continuous variables with normal distribution were presented as a mean ± standard deviation, and continuous variables deviated from a normal distribution were presented as the median (25th and 75th percentiles). Categorical variables were presented as frequency and percentage. Statistical analyses of continuous variables were conducted using a two-tailed t-test for unpaired samples or a non-parametric method, as appropriate, depending on the distribution of the data. For statistical analyses of categorical variables, the chi-square test was used. In cases of multiple comparisons, Bonferroni correction was used. A receiver operating characteristics (ROC) curve with Youden criteria was used to determine the optimal eGFR cutoff value to predict mortality. The associations between predictors and outcomes were tested by multivariate logistic regression models. The significance of the association was expressed as the odds ratio with the 95% confidence interval. Statistical analysis was performed using SAS 9.4 (SAS Institute Inc, Cary, NC, USA)

## Results

### Demographic and laboratory characteristics of patients with pneumonia

The present study included 1554 patients hospitalized with pneumonia, of whom 63.5% were males, and the mean age was 76.1±16.7 years. Overall, 79 (5.1%) patients had ESRD and 162 (10.4%) were deceased at the end of hospitalization. The mortality group had a significantly older age, as well as more patients with CKD, AKI, acute-on-chronic kidney injury and ESRD. Notably, the patients with DM, CHF, and COPD were not significantly different between the survival group and the mortality group. Biochemistry panels revealed significantly higher serum blood urea nitrite, creatinine, aspartate aminotransferase (AST), and alanine aminotransferase (ALT) and significantly lower serum albumin in the mortality group. In addition, the mortality group exhibited significantly higher white blood cell (WBC) counts, lower lymphocyte counts, and higher NLR and MLR values. The neutrophil, monocyte, and platelet counts were similar between the two groups. The mortality group was also associated with an increased number of bacteremia, ventilator use, ICU admission events, longer hospital durations, as well as higher SMART-COP and CURB65 scores (Table 1).

**Table 1:** Demographic and laboratory characteristics of patients hospitalized with pneumonia.

Characteristics	Total	Survival	Mortality	*p* value
	n = 1554	n = 1392	n = 162	
Male	987 (63.5%)	877 (63.0%)	110 (67.9%)	0.220
Age (years)	76.1 ± 16.7	75.3 ± 17.0	83.1 ± 11.2	< 0.001
CKD	441 (28.4%)	376 (27.0%)	65 (40.1%)	<0.001
AKI	298 (19.2%)	242 (17.4%)	56 (34.6%)	< 0.001
ACKI	114 (7.3%)	91 (6.5%)	23 (14.2%)	<0.001
ESRD	79 (5.1%)	65 (4.7%)	14 (8.7%)	0.028
DM	110 (7.1%)	104 (7.5%)	6 (3.7%)	0.077
CHF	111 (7.1%)	96 (6.9%)	15 (9.3%)	0.269
COPD	102 (6.6%)	93 (6.7%)	9 (5.6%)	0.584
BUN (mg/dL)	20 (13, 32)	19 (13, 29)	37 (22, 61)	< 0.001ǂ
Creatinine (mg/dL)	0.9 (0.7, 1.4)	0.9 (0.7, 1.3)	1.3 (0.8, 2.3)	< 0.001ǂ
eGFR (mL/min/1.73 m^2^)	77.1 (48.6, 93.6)	78.5 (52.8, 94.5)	53.0 (26.7, 81.9)	< 0.001ǂ
AST (U/L)	25 (19, 35)	24 (19, 33)	33 (22, 51)	< 0.001ǂ
ALT (U/L)	19 (13, 29)	18 (13, 28)	23 (14, 43)	< 0.001ǂ
Albumin (g/dL)	2.9(2.5, 3.3)	3.0(2.6, 3.4)	2.5(2.2, 2.9)	< 0.001ǂ
Hemoglobin (g/dL)	11.8±2.2	11.9±2.1	10.6±2.5	< 0.001
WBC (10^3^/mm^3^)	12.7 ± 6.2	12.6 ± 6.0	13.9 ± 8.1	0.035
Neutrophil (cell/mm^3^)	9375 (6315, 13022)	9297 (6306, 12927)	9909 (6585, 14425)	0.132ǂ
Lymphocyte (cell/mm^3^)	1080 (642, 1618)	1100 (669, 1638)	836 (458, 1443)	< 0.001ǂ
Monocyte (cell/mm^3^)	679 (424, 1013)	685 (433, 1010)	629 (377, 1067)	0.390ǂ
NLR	8.5 (4.9, 15.4)	8.4 (4.9, 14.8)	10.6 (5.2, 24.9)	<0.004ǂ
MLR	0.6 (0.4, 1.0)	0.6 (0.4, 1.0)	0.7 (0.4, 1.3)	0.026ǂ
Platelet (10^3^/mm^3^)	211 (160, 277)	209 (161, 278.5)	226 (134, 276)	0.607ǂ
Bacteremia	91 (5.9%)	72 (5.2%)	19 (11.7%)	< 0.001
Ventilator use	271 (17.4%)	190 (13.7%)	81 (50.0%)	< 0.001
ICU admission	238 (15.3%)	182 (13.1%)	56 (34.6%)	< 0.001
Hospital days (days)	11 (7, 18)	10 (7, 17)	15 (6, 30)	< 0.002ǂ
SMART-COP score	3 (2, 4)	2 (2, 3)	4 (3, 6)	<0.001ǂ
CURB65 score	2 (1, 2)	2 (1, 2)	2 (2, 3)	<0.001ǂ

ǂby Exact Wilcoxon two-sample test.

CKD, chronic kidney disease; AKI, acute kidney injury; ACKI, acute-on-chronic kidney injury; DM, diabetes mellitus; CHF, congestive heart failure; COPD, chronic obstructive pulmonary disease; ICU, intensive care unit; BUN, blood urea nitrite; eGFR, estimated glomerular filtration rate; WBC, white blood cell count; NLR, neutrophil-to-lymphocyte ratio; MLR, monocyte-to-lymphocyte ratio; AST, aspartate aminotransferase; ALT, alanine aminotransferase. Continuous variables with normal distribution were expressed as mean ± standard deviation, while those deviated from normal distribution were expressed as medium (1^st^, 3^rd^ quartile).

**Fig 1:**
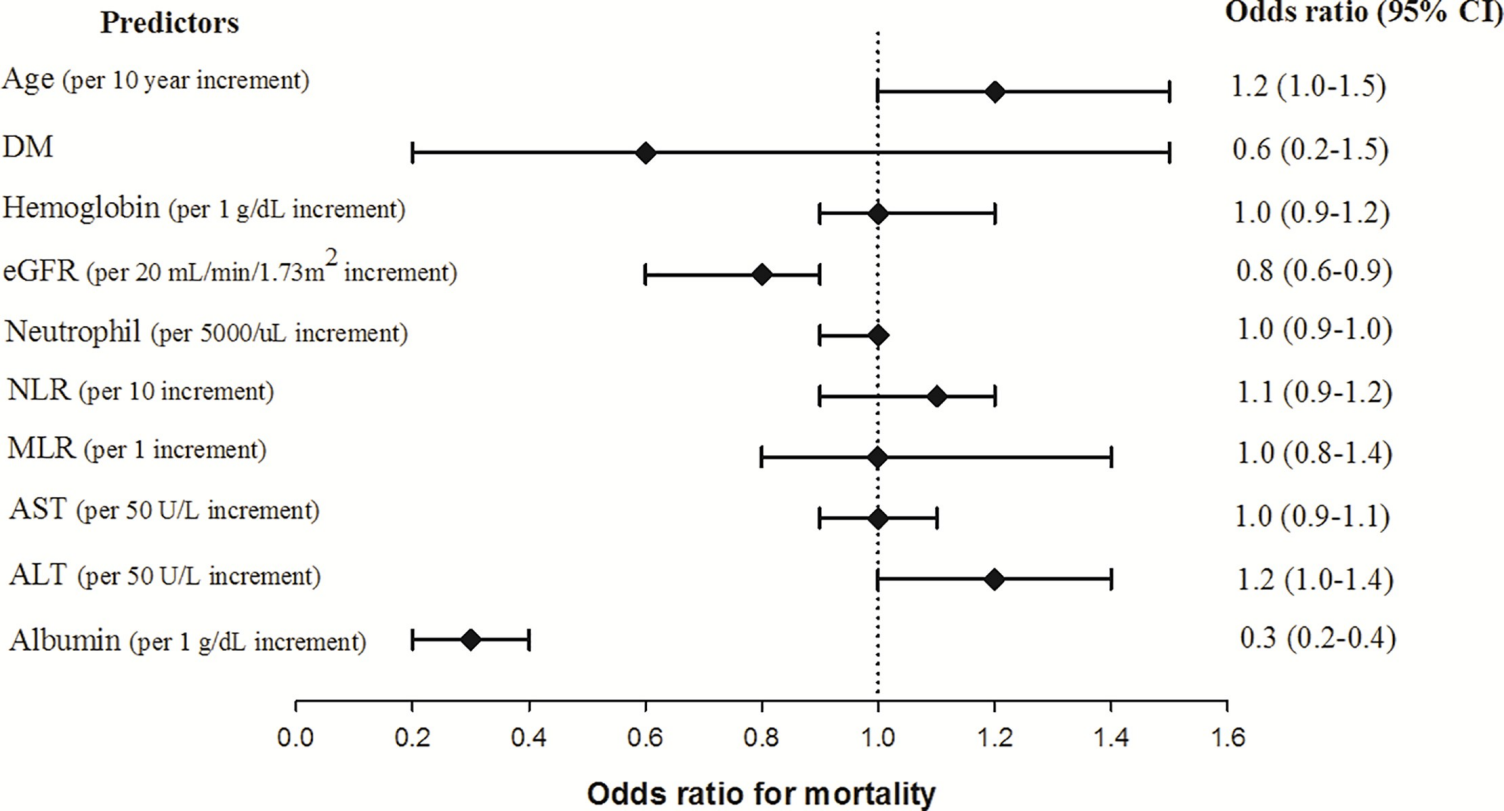
Risk factors for in-hospital mortality in patients with pneumonia. Odds ratio for mortality was calculated by multivariate logistic regression. DM, diabetes mellitus; eGFR, estimated glomerular filtration rate; NLR, neutrophil-to-lymphocyte ratio; MLR, monocyte-to-lymphocyte ratio; AST, aspartate aminotransferase; ALT, alanine aminotransferase, CI, confidence interval.

Based on the above findings, the potential risk factors for mortality were tested by logistic regression. The predictors with *p*-values < 0.2 based on univariate logistic regression, including age, DM, hemoglobin, neutrophil count, NLR, MLR, serum AST, ALT, and albumin levels, were included in multivariate analyses. Based on the multivariate logistic regression model, only eGFR and serum albumin levels remained significantly associated with mortality, indicating the important effect of eGFR on pneumonia patient outcome (Fig 1).

### Microbiological analysis of sputum samples from pneumonia patients and relationship with renal function

As eGFR was shown to be significantly associated with mortality for patients hospitalized with pneumonia, an ROC curve with Youden criteria was used to determine the optimal eGFR cut-off value to predict this event. The ROC curve had an area under the curve of 0.66, and the Youden criteria showed that eGFR of 55 mL/min/1.73 m^2^ was the optimal cut-off value to predict mortality.

To investigate the relationship between renal function and pneumonia-related mortality, microbiological analyses of sputum from patients with eGFR ≥ 55 and <55 mL/min/1.73m^2^ was performed. Notably, only 1371 patients with available microbiology reports were included. Overall, the microbial profiles from the sputum samples of the two groups were significantly different (P < 0.001 based on a chi-square test). The events of gram-negative bacillus (GNB) and gram-positive coccus (GPC) were not significantly different between the two groups (significance defined by Bonferroni correction as *p* < 0.006 and < 0.017 for each specific pathogen of GNB and GPC, respectively). The group with eGFR < 55 mL/min/1.73 m^2^ had a significantly increased incidence of fungi and a decreased incidence of mixed anaerobics in their sputum. Pathogen-specific analysis showed that the group with eGFR < 55 mL/min/1.73 m^2^ had a significantly lower incidence of *Pseudomonas aeruginosa* (*Ps*. *aeruginosa*), but a significantly increased incidence *Escherichia coli*, *Klebsiella pneumoniae* and *S*. *aureus* (Table 2).

**Table 2:** Sputum microbiological analysis of patients hospitalized with pneumonia categorized by eGFR.

Characteristics	Total (n = 1371)	eGFR≧55 (n = 963)	eGFR<55 (n = 408)	*p* value
**GNB**				
*Ps*. *aeruginosa*	207 (15.1%)	163 (17.0%)	44 (10.8%)	0.004
*Kl*. *pneumoniae*	185 (13.5%)	115 (11.9%)	70 (17.2%)	0.010
*Acinetobacter spp*.	67 (4.9%)	45 (4.7%)	22 (5.4%)	0.572
*Escherichia coli*	62 (4.5%)	36 (3.7%)	26 (6.4%)	0.032
*Proteus spp*.	45 (3.3%)	37 (3.8%)	8 (2.0%)	0.074
*Haemophilus spp*.	40 (2.9%)	33 (3.4%)	7 (1.7%)	0.085
*Serratia spp*.	23 (1.7%)	19 (2.0%)	4 (1.0%)	0.191
*Stenotrophomonas spp*.	22 (1.6%)	18 (1.9%)	4 (1.0%)	0.231
**GPC**				
*S*. *aureus*	105 (7.7%)	62 (6.4%)	43 (10.5%)	0.009
*Strep*. *pneumoniae*	11 (0.8%)	7 (0.7%)	4 (1.0%)	0.741ǂ
*Strep*. *Spp*.	43 (3.1%)	31 (3.2%)	12 (2.9%)	0.787
**Mixed anaerobic**	140 (10.2%)	111 (11.5%)	29 (7.1%)	0.014
**Fungi**	39 (2.8%)	21 (2.2%)	18 (4.4%)	0.023
**No growth**	579 (42.2%)	408 (42.4%)	171 (41.9%)	0.876

ǂby Fisher’s Exact test.

eGFR, estimated glomerular filtration rate; GNB, gram-negative bacillus; *Ps*. *aeruginosa*, *Pseudomonas aeruginosa*; *Kl*. *pneumoniae*, *Klebsiella pneumoniae*; GPC, gram-positive coccus; *S*. *aureus*, *Staphylococcus aureus*; *Strep*., *Streptococcus*; WBC, white blood cell; NLR, neutrophil-to-lymphocyte ratio; MLR, monocyte-to-lymphocyte ratio.

**Fig 2:**
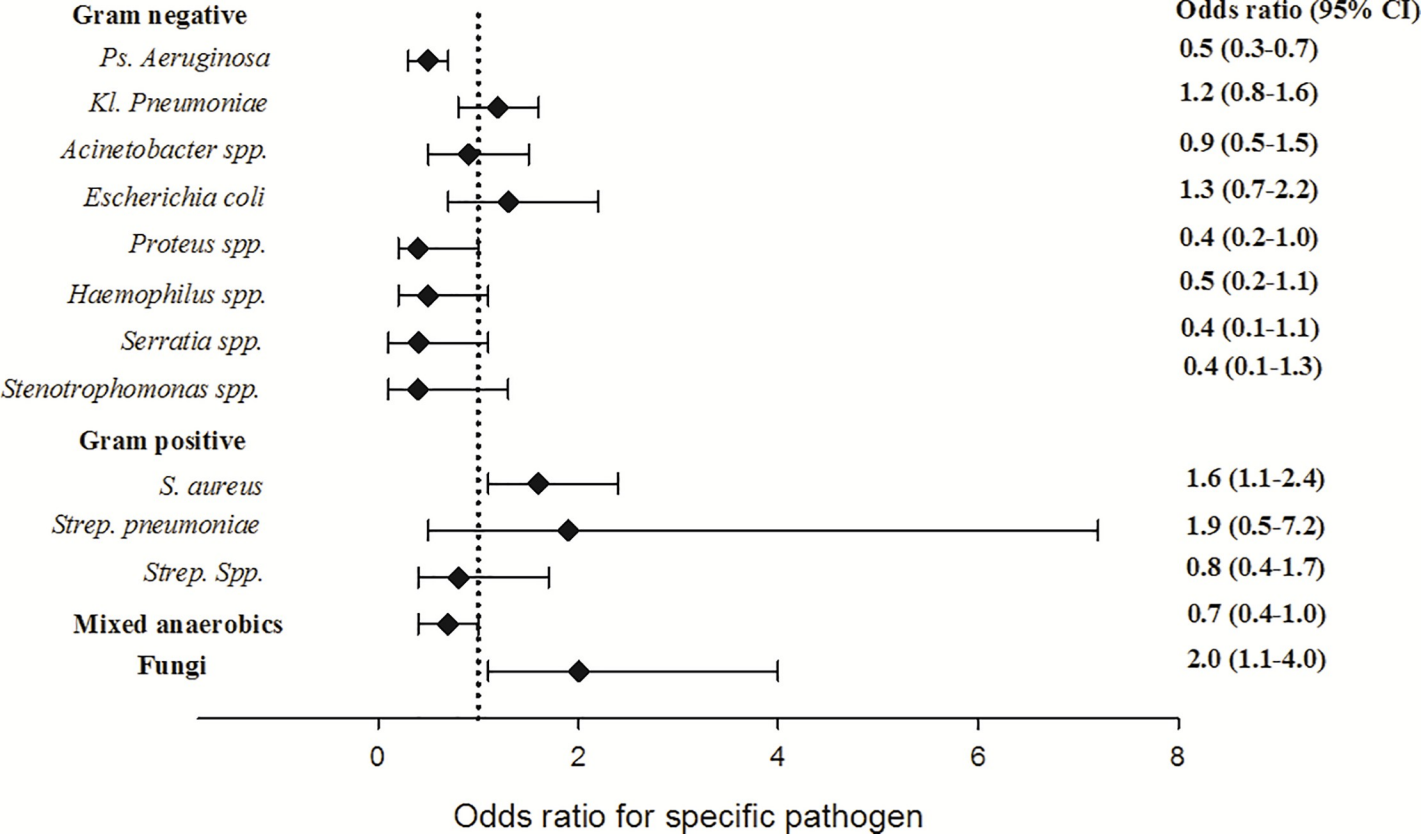
Risk of isolation of specific pathogens in patients with eGFR of < 55.3 mL/min/1.73 m^2^. Odds ratios for specific pathogens were calculated using a multivariate logistic regression model adjusted by age and gender. CI, confidence interval; eGFR, estimated glomerular filtration rate; *Ps*. *aeruginosa*, *Pseudomonas aeruginosa*; *Kl*. *pneumoniae*, *Klebsiella pneumoniae*; *S*. *aureus*, *Staphylococcus aureus*; *Strep*., *Streptococcus*.

Logistic regression was used to confirm the association between eGFR and microbiologic characteristics. Multivariate (adjusted by age and gender) logistic regression analyses showed that patients with eGFR < 55 mL/min/1.73 m^2^ were at a significantly higher risk of fungi and a lower risk of mixed anaerobics. Pathogen-specific analysis showed that patients with eGFR < 55 mL/min/1.73m^2^ were at a significantly lower risk of *P*. *aeruginosa* and a significantly higher risk of *S*. *aureus* (Fig 2).

The above findings indicated that patients with decreased renal function were at increased risk of *S*. *aureus* and fungi in the sputum, which might underlie the association between decreased renal function and pneumonia-related mortality.

### Immune cell profiles and outcome of pneumonia patients with and without decreased eGFR

The group with eGFR < 55 mL/min/1.73 m^2^ had significantly lower platelet, basophil, eosinophil, and lymphocyte counts, as well as significantly higher NLR and MLR. Otherwise, WBC, neutrophil, and monocyte counts were similar between the two groups (Table 3).

**Table 3:** The immune cell profiles of patients hospitalized with pneumonia categorized by eGFR.

Immune cells	Total (n = 1371)	eGFR ≥ 55 (n = 963)	eGFR < 55 (n = 408)	*p* value
WBC (10^3^/mm^3^)	12.8 ± 6.4	12.6 ± 6.1	13.1 ± 7.0	0.215
Platelet (10^3^/mm^3^)	213 (161, 279)	221 (171, 290)	193.5 (150, 251)	<0.001ǂ
Neutrophil (cell/mm^3^)	9418 (6315, 13422)	9287 (6270, 12935)	9568 (6413, 13984)	0.139
Eosinophil (cell/mm^3^)	32.6 (0, 130.1)	3.8 (0, 143.3)	21.3 (0, 96.1)	0.001ǂ
Basophil (cell/mm^3^)	22 (0, 48)	24 (0, 52)	16 (0, 42)	<0.001ǂ
Lymphocyte (cell/mm^3^)	1080 (642, 1623)	1114 (685, 1689)	944 (545, 1425)	<0.001ǂ
Monocyte (cell/mm^3^)	675 (426, 1013)	676 (438, 1015)	675 (394, 1012)	0.438ǂ
NLR	8.6 (4.9, 15.3)	8.2 (4.7, 14.3)	9.9 (5.3, 18.2)	<0.001ǂ
MLR	0.6 (0.4, 1.0)	0.6 (0.4, 1.0)	0.7 (0.4, 1.2)	0.001ǂ

ǂby Exact Wilcoxon two-sample test.

eGFR, estimated glomerular filtration rate; WBC, white blood cell; NLR, neutrophil-to-lymphocyte ratio; MLR, monocyte-to-lymphocyte ratio. Continuous variables with normal distribution were expressed as mean ± standard deviation, while those deviated from normal distribution were expressed as medium (1^st^, 3^rd^ quartile).

**Fig 3:**
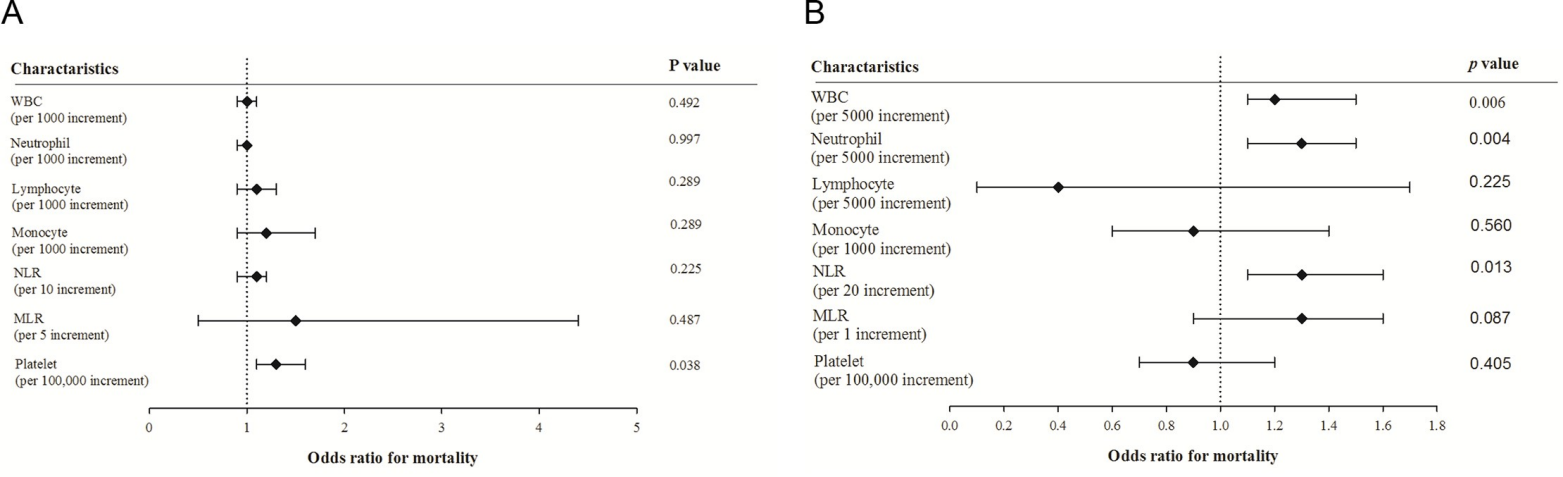
The association between immune cell profiles and mortality in patients hospitalized with pneumonia. A. The odds ratios for mortality in patients with eGFR ≥ 55 mL/min/1.73 m^2^. B. The odds ratios for mortality in patients with eGFR < 55 mL/min/1.73 m^2^. WBC, white blood cell; NLR, neutrophil-to-lymphocyte ratio; MLR, monocyte-to-lymphocyte ratio.

As features of immune cell profiles differ between renal function groups, logistic regression was used to confirm the association between immune cell profiles and mortality in patients of each renal function group. In patients with eGFR ≥ 55 mL/min/1.73 m^2^, only higher platelet counts were significantly associated with mortality (Fig 3A). In contrast, in patients with eGFR < 55 mL/min/1.73 m^2^, both higher WBC counts, neutrophil counts and NLR were significantly associated with higher pneumonia-related mortality (Fig 3B). The above findings identified different immune cell profiles and their association with pneumonia outcome in patients with and without decreased renal function.

### Characteristics and outcomes of patients with S. aureus infection

Patients with *S*. *aureus* infection were significantly older with a higher percentage of CKD and acute-on-chronic kidney injury. In addition, significantly higher blood urea nitrite, lower eGFR, lower serum albumin, lower lymphocyte counts, and higher NLR were identified in patients with *S*. *aureus infection*. Patients with *S*. *aureus* infection were also associated with increased ventilator use and ICU admissions, a longer period of hospitalization, higher mortality, and higher CURB65 score. Notably, the SMART-COP score, a pneumonia severity score without consideration of renal function, was similar between patients with or without *S*. *aureus* infection, implying the association between renal impairment and susceptibility to *S*. *aureus* infection. (Table 4). Of the isolated *S*. *aureus*, 60% were methicillin resistant *S*. *aureus* (*MRSA*), most of which (96.8%) were not sensitive to empirical treatment. However, patients with MRSA did not have more mortality or other adverse outcomes (Table 5). These findings indicated that in patients with pneumonia, *S*. *aureus* is associated with decreased renal function and its characteristic immune cell profile, specifically elevated NLR, which might be the cause of worse pneumonia-related outcomes in patients with decreased renal function.

**Table 4:** Characteristics and outcomes associated with *S*. *aureus* in sputum.

Characteristics	Without *S*. *aureus* (n = 1266)	With *S*. *aureus* (n = 105)	*p* value
Age (year)	76.0 ± 16.7	79.7 ± 14.8	0.029
Male	808 (63.8%)	71 (67.6%)	0.436
CKD	351 (27.7%)	43 (41.0%)	0.004
AKI	230 (18.2%)	22 (21.0%)	0.479
ACKI	80 (6.3%)	13 (12.4%)	0.018
DM	85 (6.7%)	7 (6.7%)	0.985
CHF	92 (7.3%)	3 (2.7%)	0.087
COPD	94 (7.4%)	5 (4.8%)	0.311
Hemoglobin (g/dL)	11.8 ± 2.2	11.6 ± 2.2	0.234
BUN (mg/dL)	20 (13, 31)	27 (16, 46)	< 0.001ǂ
Creatinine (mg/dL)	0.9 (0.7, 1.3)	1.0 (0.7, 1.6)	0.105ǂ
eGFR (mL/min/m^2^)	78.2 (50.7, 93.9)	69.2 (40.5, 86.3)	0.020ǂ
AST (U/L)	25 (19, 35)	25 (19, 37)	0.294ǂ
ALT (U/L)	18 (12, 29)	21.5 (13, 33)	0.076ǂ
Albumin (g/dL)	2.9 (2.5, 3.3)	2.7 (2.5, 2.9)	< 0.003ǂ
WBC (10^3^/mm^3^)	12.7 ± 6.2	13.6 ± 7.7	0.221
Neutrophil (cell/mm^3^)	9360 (6310, 13163)	9809 (6488, 15059)	0.159ǂ
Lymphocyte (cell/mm^3^)	1094 (652, 1644)	810 (500, 1339)	0.004ǂ
Monocyte (cell/mm^3^)	685 (437, 1014)	592 (330, 963)	0.074ǂ
NLR	8.4 (4.8, 15.0)	10.5 (6.9, 21.2)	0.002ǂ
MLR	0.6 (0.4, 1.0)	0.7 (0.4, 1.2)	0.251ǂ
Bacteremia	68 (5.4%)	8 (7.6%)	0.333
Ventilator use	229 (18.1%)	28 (26.7%)	0.030
ICU admission	198 (15.6%)	29 (27.6%)	0.002
Hospital days (day)	11 (7, 18)	14 (10, 25)	< 0.010ǂ
Mortality	128 (10.1%)	19 (18.1%)	0.011
SMART-COP	3 (2, 4)	3 (2, 4)	0.255
CURB65	2 (1, 2)	2 (1, 3)	<0.001

ǂby Exact Wilcoxon two-sample test.

*S*. *aureus*, *Staphylococcus aureus*; CKD, chronic kidney disease; AKI, acute kidney injury; ACKI, acute-on-chronic kidney injury; DM, diabetes mellitus; CHF, congestive heart failure; COPD, chronic obstructive pulmonary disease; BUN, blood urea nitrite; eGFR, estimated glomerular filtration rate; WBC, white blood count; NLR, neutrophil-to-lymphocyte ratio; MLR, monocyte-to-lymphocyte ratio; AST, aspartate aminotransferase; ALT, alanine aminotransferase; ICU, intensive care unit. Continuous variables with normal distribution were expressed as mean ± standard deviation, while those deviated from normal distribution were expressed as medium (1^st^, 3^rd^ quartile).

**Table 5:** Resistance to methicillin of *S*. *aureus* and the outcomes of pneumonia patients.

Character	*MSSA*	*MRSA*	P value
Number	42 (40.0%)	63 (60.0%)	n/a
Age (year)	79.8±15.3	79.7±14.5	0.96
Male	23 (54.8%)	48 (76.2%)	0.02
CKD	15 (35.7%)	28 (44.4%)	0.37
AKI	7 (16.7%)	15 (23.8%)	0.38
eGFR (mL/min/1.73m^2^)	78.5 (50.1, 86.6)	62.1 (26.8, 85.3)	0.22
Empirical treatment			n/a
Piperacillin/tazobactam	14 (33.3%)	28 (44.4%)	
Amoxicillin/clavulanate	10 (23.8%)	6 (9.6%)	
2^nd^ cephalosporines	0 (0%)	1 (1.6%)	
3^rd^ cephalosporines	5 (11.9%)	8 (12.7%)	
4^th^ cephalosporines	1 (2.4%)	0 (0%)	
Quinolones	9 (21.4%)	9 (14.3%)	
Carbapenems	3 (7.1%)	11 (17.5%)	
Teioplanin	1 (2.4%)	1 (1.6%)	
Inappropriate empirical treatment	2 (4.8%)	61 (96.8%)	<0.01
Hospital days (day)	13.5 (10, 24)	15 (10, 26)	0.91
ICU admission	10 (23.8%)	19 (30.2%)	0.48
Ventilator	13 (31.0%)	15 (23.8%)	0.42
Mortality	10 (23.8%)	9 (14.3%)	0.21

*S*. *aureus*, *Staphylococcus aureus*; *MSSA*, *methicillin-sensitive Staphylococcus aureus*; *MRSA*, *methicillin-resistant Staphylococcus aureus*; CKD, chronic kidney disease; AKI, acute kidney injury; eGFR, estimated glomerular filtration rate; ICU, intensive care unit. Continuous variables with normal distribution were expressed as mean ± standard deviation, while those deviated from normal distribution were expressed as medium (1^st^, 3^rd^ quartile).

## Discussion

The main findings of the present study included a higher risk of *S*. *aureus* infection, and higher NLR in pneumonia patients with eGFR < 55 mL/min/1.73 m^2^. Furthermore, in pneumonia patients with *S*. *aureus* infection, lower eGFR, lymphocyte counts, and higher NLR were observed. It is plausible that decreased eGFR is associated with higher pneumonia-related mortality through disturbed immunity and vulnerability to *S*. *aureus* infection. Notably, in clinical practice, fungi in sputum microbiological samples are more often interpreted as colonization. Therefore, our analysis focused on *S*. *aureus*.

We preliminarily revealed higher risk of *S*. *aureus* infection in pneumonia patients with decreased renal function. Previously, a prospective observational study in Spain showed that *Streptococcus pneumoniae* was the most frequent cause of pneumonia in patients with CKD. Additionally, the overall etiology of pneumonia and the occurrence of *S*. *aureus* in patients with CKD were similar to those in individuals without CKD [32]. A possible explanation for the discrepancy between this previous study and the present study might be that vaccination for pneumococci has been universally provided to elderly (≥ 75 years of age) since 2007 and newborns since the beginning of 2015 in Taiwan. In 2005, two small-scale studies showed that *S*. *pneumoniae* was the most frequent causative pathogen of pneumonia in Taiwan, contributing to 24–26% of cases [33–34]. It is thus reasonable that pneumococci vaccination has caused a decline in the incidence of *S*. *pneumoniae* infection in Taiwan.

The present single-center study in Taiwan revealed microbiologic features of pneumonia that deviate from most published reports [35], showing increased significance of gram-negative bacilli and *S*. *aureus* and a decreased role for *S*. *pneumoniae*. To date, a nationwide analysis of the pathogenic etiologies of pneumonia in Taiwan has not been performed; thus, we were unable to compare with national microbiological data between these studies. One possible explanation for the microbiology results might be that the study site was located in a community comprising an aged population and numerous healthcare facilities, which leads to a higher frequency of healthcare-associated pneumonia and deviated microbiological results. Indeed, the resistance to methicillin is highly prevalent in *S*. *aureus* in the present study.

In this study, we preliminarily revealed NLR as a marker of mortality in pneumonia patients with decreased renal function. Recently, NLR has been evaluated as a predictor of outcome for various infectious diseases, which, however, provided inconsistent results [36–37]. In a study enrolling 395 patients with community-acquired pneumonia, NLR was acceptable for predicting mortality with an area under the ROC curve of 0.701 [38]. In our study, overall, the association between NLR and mortality was insignificant. Nonetheless, based on subgroup analysis of patients with eGFR < 55 mL/min/1.73 m^2^, NLR was significantly associated with mortality of pneumonia, which remained insignificant in the group with eGFR ≥ 55 mL/min/1.73 m^2^. This indicated a potential connection between elevated NLR, decreased renal function, and mortality of pneumonia. However, the underlying disturbance of immunity in patients with decreased renal function remains to be investigated.

The other preliminary finding of this study was that pneumonia patients with *S*. *aureus* infection exhibited significantly higher NLR than those without, implying an association between higher NLR and *S*. *aureus* infection. In support to our finding, a study enrolling 121 patients with infective endocarditis, of which *Staphylococcus* was the most frequent pathogen (33%), NLR > 7.1 predicted adverse outcomes [38]. Nonetheless, solid evidence suggesting a causative relationship between NLR and susceptibility to *S*. *aureus* infection has not been presented.

The limitations of the present study, as previously stated, included the single center design, such that the microbiology results might have been affected by regional epidemics. Another limitation is the retrospective design and inability to control potential some confounding factors, including prior antibiotic use. In addition, in this study, community-acquired pneumonia and healthcare-associated pneumonia were not considered separately, which might also affect the results. In this study, we used the results of sputum cultures for microbiology analysis, which are usually considered as likely cause of pneumonia. However, since bronchoscopy is not routinely performed in patients with pneumonia, the more reliable bronchial aspirate culture could not be discussed extensively in the present study. Finally, there was only a small number of ESRD patients in the present study that a separated analysis for pathogen in this group of patients is not feasible. The strength of the present study included the relatively large number of cases and the complete microbiology data. Notably, despite the significant role of *S*. *aureus* in pneumonia patients with decreased eGFR, gram-negative bacilli (*P*. *aeruginosa* and *K*. *pneumoniae*) also contributed significantly to pneumonia in this population.

## Conclusions

This study showed a significantly higher risk of *S*. *aureus* in pneumonia patients with decreased renal function, which might cause higher pneumonia-related mortality in this population. Moreover, both in patients with decreased renal function and in patients with *S*. *aureus*, higher NLR was detected, implying an association between higher NLR and susceptibility to *S*. *aureus* infection. However, the causative relationship remains to be defined.
